# Oral squamous cell carcinoma (OSCC)-derived exosomal MiR-221 targets and regulates phosphoinositide-3-kinase regulatory subunit 1 (PIK3R1) to promote human umbilical vein endothelial cells migration and tube formation

**DOI:** 10.1080/21655979.2021.1932222

**Published:** 2021-06-08

**Authors:** Shuqi He, Wu Zhang, Xian Li, Junjie Wang, Xufeng Chen, Yu Chen, Renfa Lai

**Affiliations:** a Medical Centre of Stomatology, The First Affiliated Hospital of Jinan University, Guangzhou, China; b Department of Oral & Maxillofacial Surgery, First Affiliated Hospital of Sun Yat-Sen University, Guangzhou, China

**Keywords:** Oral squamous cell carcinoma (OSCC), exosome, miR-221, PIK3R1, HUVEC, migration, tube formation

## Abstract

Oral squamous cell carcinoma (OSCC) is the most common tumor of the oral cavity. Studies have shown that exosomal miRNAs from cancer cells play an important role in mediating the cellular environment. The objective was to investigate the effect of OSCC-derived exosomes microRNA-221 (miR-221) in OSCC. We used quantitative real-time PCR (qRT-PCR) and western blotting to determine PIK3R1 and miR-221 expressions in OSCC tissue or peripheral blood serum. Exosomes of OSCC cell line CAL27 were extracted and characterized. Exosomal miR-221 expression was detected by qRT-PCR. Dual-luciferase was performed to validate the targeted regulatory relationship of miR-221 on PIK3R1. Transwell and tube formation assay were applied to detect the effect of OSCC-derived exosomal miR-221 on HUVEC migration and angiogenesis. qRT-PCR confirmed that PIK3R1 expression was downregulated in OSCC tissue and cell line, while miR-221 expression was upregulated. miR-221 expression in OSCC cell line-derived exosome elevated. miR-221 could target and negatively regulate PIK3R1 expression. In addition, OSCC-derived miR-221 could promote HUVEC migration and angiogenesis. In conclusion, OSCC-derived exosomal miR-221 could target and negatively regulate PIK3R1 expression, as well as promote vascular endothelial cell migration and angiogenesis.

## GRAPHICAL ABSTRACT

**Figure:**
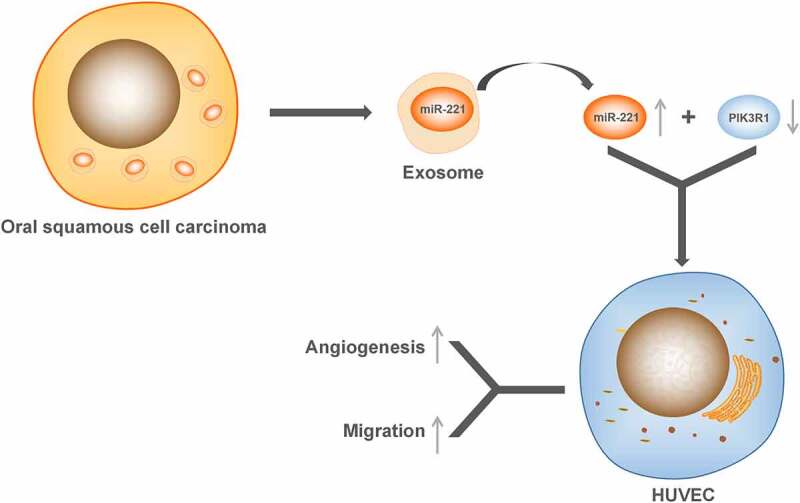

## Introduction

Oral squamous cell carcinoma (OSCC) is the most frequently seen tumor in the oral cavity and has become a significant public health problem, with a 5-year survival rate of 50–60% only [1]. So far, OSCC treatment is still dependent on surgical resection, supplemented by radiotherapy and chemotherapy, emphasizing the important role of radical and complete treatments in the prognosis of patients. However, for patients with advanced tumors and recurrence, the five-year survival rate has no significant increase in recent years regardless of the continuous improvement in surgical methods and adjuvant therapy [2]. Biomarkers, which are indicators of the physiological status and changes of cells during the disease progression, have unique advantages in accurately and sensitively evaluating early and mild damage and can be used for early diagnosis and prognosis indicator in the treatment of OSCC, so it is important to find cancer markers with the accurate diagnostic and prognostic abilities for OSCC, especially at an early stage [3–5]. In view of the potential in clinical application of biomarkers, the search for specific early tumor markers for OSCC has become one of the major research hotspots in recent years.

Phosphoinositide-3-kinase regulatory subunit 1 (PIK3R1) is abnormally regulated in various malignancies, and is correlated to poor prognosis. CircAKT3 can promote the expression of PIK3R1 through competitive binding to miR-198, followed by promoting cisplatin resistance in gastric cancer [6]. In node-positive pancreatic ductal adenocarcinoma (PDAC), circNFIB1 can competitively bind to miR-486-5p and thus upregulate PIK3R1 expression, and to further downregulate VEGF-C expression by inhibiting the PI3K/Akt pathway, finally inhibiting lymphangiogenesis and PDAC lymph node metastasis [7]. It can be seen that PIK3R1 plays a dual role in tumors, either as a tumor suppressor or as a tumor promoter participating in tumor development. Since the expression and function of PIK3R1 in OSCC remain unclear, we would investigate PIK3R1 expression in OSCC.

Exosomes are naturally secreted vesicae (diameter 40–150 nm) from cells, which consist of a series of protein, lipid, mRNAs and microRNAs [8]. Because of its ability to stably deliver drugs, therapeutic microRNAs as well as proteins, etc., exosomes can be used as potential tumor-targeted drug carriers [9]. MicroRNAs (miRNAs) are single-stranded non-coding RNAs, around 18–23 nt in length, which involve in regulating gene expressions at the posttranscriptional level [10]. MiRNAs cannot directly enter the local microenvironment or humoral circulation, and their transmission between cells depends on vehicles or direct contact of cells. Exosomes can maintain structural stability in the microenvironment and humoral circulation, prevent degradation of contents, and are ideal carrier for miRNA transmission between cells and participation in humoral circulation [11]. Exosomes that are released by tumor cells contain lots of abnormal miRNAs, which are important in encouraging tumor proliferation, invasion and metastasis [12]. It has been proven that an upregulation of microRNA-221 (miR-221) could be detected in exosomes secreted by a variety of malignant cells, promoting an malignant phenotype in tumors [13–15]. However, there are no studies in the literature on the relevant role of OSCC-derived exosomal miR-221 in OSCC. Here, we investigated the effect of exosomal miR-221 in OSCC. And we found that miR-221 expression was upregulated in exosomes secreted by OSCC cells, and promote HUVEC migration angiogenesis. These findings suggested that miR-221/PIK3R1 could be a prospective target for clinical OSCC treatment.

## Material and methods

### Human tissue samples

From June 2018 to November 2019, 16 patients with OSCC who received maxillofacial surgeries and 16 healthy control samples at the First Affiliated Hospital of Jinan University were selected. None of the selected patients received radiotherapy or chemotherapy. Healthy control samples did not have medical history such as hypertension, diabetes, coronary heart disease, pulmonary disease, or malignancy. Informed consent was obtained from all individuals, and the research protocols were approved by the Ethics Committee of the First Affiliated Hospital of Jinan University.

### qRT-PCR

Total RNA was extracted with TRIzol reagent (Invitrogen, Carlsbad, CA, USA) from collected samples. A reverse transcription kit (TaKaRa Bio., Tokyo, Japan) was used to reverse transcribe according to the instructions. Gene expression was detected using a LightCycler480 quantitative fluorescence PCR instrument (Roche Diagnostics, Indianapolis, IN, USA), and conditions were set to the operation instructions of the quantitative fluorescence PCR kit (SYBR Green Master, Roche Diagnostics). Parameters for thermal cycling were: 95°C for 10 s, 45 cycles of 95°C for 5 s, 60°C for 10 s, and 72°C for 10 s; the final extension was at 72°C for 5 min. Three replicates per reaction were set up for quantitative PCR. GAPDH and U6 were used for internal reference. Data were analyzed with the 2^−ΔΔCt^ method. The amplification primer sequences are listed in Table 1.

**Table 1.** Sequences of amplification primer

Gene		Sequence (5'–3')
*miR-221*	Forward	ACCTGGCATACAATGTAG
	Reverse	GAACATGTCTGCGTATCTC
*U6*	Forward	UUCUCCGAACGUGUCACGUTT
	Reverse	UGACACGUUCGGAGAATT
*PIK3R1*	Forward	CGCCTCTTCTTATCAAGCTCGTG
	Reverse	GAAGCTGTCGTAATTCTGCCAGG
*GAPDH*	Forward	GGAGCGAGATCCCTCCAAAAT
	Reverse	GGCTGTTGTCATACTTCTCATGG

## Western blot

Cell lysis was done using RIPA lysis solution (Beyotime, Shanghai, China) to acquire protein. BCA kit (Beyotime) was used to measure the protein concentration. Proteins were separated by SDS-PAGE and then transferred to PVDF membrane. The membranes were blocked with 5% nonfat milk for 1 h at room temperature, and subsequently incubated with primary antibody GAPDH (5174S, 1:1000, Cell Signaling Technology, Danvers, USA), PIK3R1 (4257S, 1:1000, Cell Signaling Technology), Tsg101 (ab125011, 1:1000, Abcam, MA, USA), Alix (92880S, 1:1000, Cell Signaling Technology), CD63 (55051S, 1:1000, Cell Signaling Technology) at 4°C overnight. Membranes were treated with secondary antibody (horseradish peroxidase-labeled goat anti-rabbit IgG, 1:5000, Beijing ComWin Biotech Co., Ltd., China, Beijing) for 1 h at room temperature. After adding the developer to the membrane, examination was done by a chemiluminescence imaging system (Bio-rad).

### Cell culture

Human umbilical vein endothelial cells (HUVECs), normal epithelial human oral keratinocyte (HOK) cell line and human tongue squamous cell carcinoma cell line CAL27 were from ATCC cell bank (Rockefeller, Maryland, USA). Cells were cultured in RPMI-1640 (Thermo Fisher Scientific, Waltham, MA, USA) supplemented with 10% fetal bovine serum (FBS; Biological Industries, Israel) and 1% penicillin-streptomycin (Solarbio, Beijing, China). All cell lines were incubated at 37°C in a humidified atmosphere of 5% CO_2_.

### Cell transfection and grouping

Both miR-221 inhibitor and its NC (100 nM) were from Gemma Gene Company (Shanghai, China). Lipofectamine 2000 reagent (Invitrogen, Carlsbad, CA, USA) was used to perform transfection according to the instructions. The cell groups were named according to their respective transfection reagents, that is, (1) NC: untransfected cells; (2) inhibitor NC: cells transfected with inhibitor NC; (3) inhibitor: with miR-221 inhibitor; (4) mimics NC: with mimics NC; (5) mimics: with miR-221 mimics; (6) exo: cultured with cal-27-derived exosomes; (7) exo+inhibitor NC: transfected with inhibitor NC and cultured with cal-27-derived exosomes; (8) exo+inhibitor: transfected with miR-221 inhibitor and cultured with cal-27-derived exosomes.

### Exosome extraction from cell supernatant

When cells grew to about 70–80%, the supernatant was discarded and cells were cultured with DMEM for 24 hours (for the purpose of excluding the exosomes in fetal bovine serum). 2000 ml of the supernatant was collected, and then the exosomes were extracted as described previously [16].

### Transmission electron microscopy (TEM)

The prepared exosome samples were drawn with a capillary pipette and dropped on a copper mesh with a supporting membrane; after a brief setting, excess liquid was removed from the edge of the liquid beads with filter paper. Three percent tungsten phosphate solution was dropped on the copper mesh and stained for 3 min, and then the staining solution was removed and the dried specimen was observed by electron microscope at 80 kV.

### Dual-luciferase report system

Online database TargetScan (http://www.targetscan.org/vert_72/) predicted the binding site of miR-221 to PIK3R1. Based on the predictions, the wild-type (WT) sequence and mutant sequence of the binding site (mut-PIK3R1, wt-PIK3R1) were designed and synthesized. The sequences were then inserted into the luciferase reporter gene vector (pGL3-Basic). Transfection groups were designed according to the purpose of the experiment, and 100 µl of cell lysate was added 48 h after transfection and was placed on a shaker at room temperature for 20 min for complete cell lysis. Firefly luciferase activity was measured by adding 50 µl of lysed cell to 50 µl of luciferase reaction solution (Promega, Madison, WI, USA). 50 µl of StopampGlo reagent (Promega) was added and Renilla luciferase activity was examined after thoroughly mixed. Renilla luciferase activity was used as an internal reference.

### HUVEC migration

HUVEC cells were transferred to the upper chamber of a Transwell coated with Matrigel, and 500 µl of complete medium containing 10% FBS was added to 24-well plate lower chamber. Following 18 hours of routine culture, cells were rinsed twice with PBS. The matrigel and cells in the upper chamber were wiped off, fixed in 4% paraformaldehyde and 0.1% crystal violet stained for 15 min. Products were observed and photographed under a microscope.

### HUVEC tube formation assay

Before the tube formation assay, Matrigel, 24-well culture plates, and pipette tips were placed at 4°C overnight to fully liquefy/precool. HUVEC culture medium was switched to serum-free medium, and HUVEC were starved for 24 hours. 250 µl Matrigel was transferred to wells of the 24-well plate and placed in a 37°C incubator for 30 min to solidfy. Cell digestion was done with 0.25% trypsin-EDTA solution; digestion was ended by 10% serum culture medium and the mixture was made into single-cell suspension, concentration adjusted to 7.5 × 10^6^ cells/ml. After mixing 300 µl serum-containing culture medium with 10 µl single-cell suspension with adjusted concentration, the mixture was added to 24-well plate one by one (3 duplicate wells for each group). Following routine culture for 10 h, photography and analysis were performed. The experiment was repeated three times.

### Data analysis

All the above procedures were repeated three times. Data were presented as mean ± standard deviation (SD). All quantitative analyses were completed using GraphPadPrism5 (GraphPad Software Inc., San Diego, CA, USA). The obtained data were statistically analyzed with SPSS 18.0 (IBM Corp., Armonk, NY, USA). To compare treatment groups and control group, one-way analysis of variance (Dunnett’s method) was used. P < 0.05 was considered statistically significant.

## Results

### Decreased PIK3R1 Expression in OSCC Tissue

**Figure 1.**
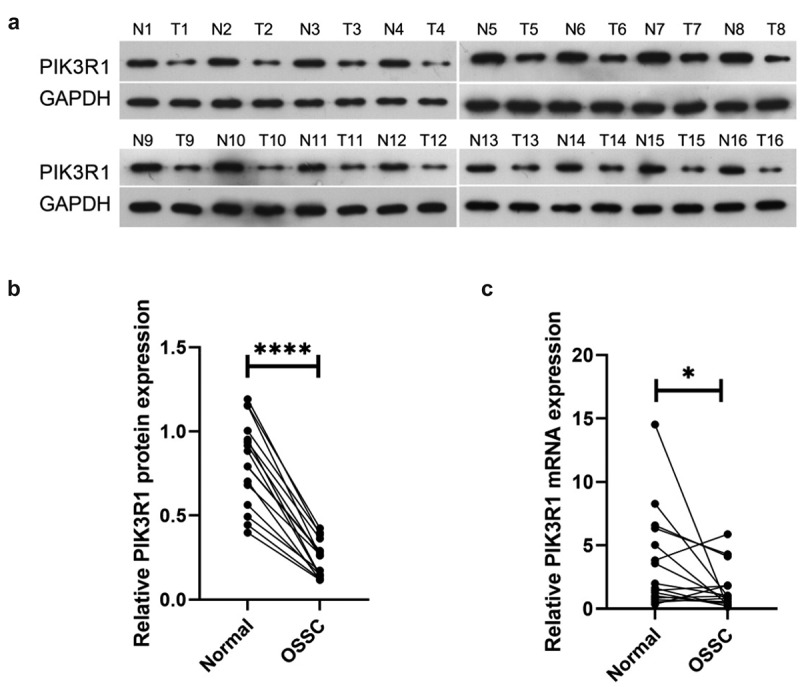
Decreased PIK3R1 expression in OSCC tissue

PIK3R1 expression levels in healthy tissues (NC) and OSCC tissues (OSCC) were examined respectively using western blot and qRT-PCR. Western blot results (Figure 1a – b) demonstrated that PIK3R1 protein expression significantly decreased in OSCC compared with NC. The qRT-PCR results (Figure 1c) revealed that PIK3R1 mRNA expression drastically decreased in OSCC in comparison to NC. The earlier results indicated that PIK3R1 expression decreased in OSCC tissues.

(a and b) Western blot monitored PIK3R1 protein expression levels in tissues of each group; (c) qRT-PCR monitored PIK3R1 mRNA expression level in each group’s tissues. *P < 0.05; ****P < 0.0001.

## Elevated MiR-221 expression in OSCC

**Figure 2.**
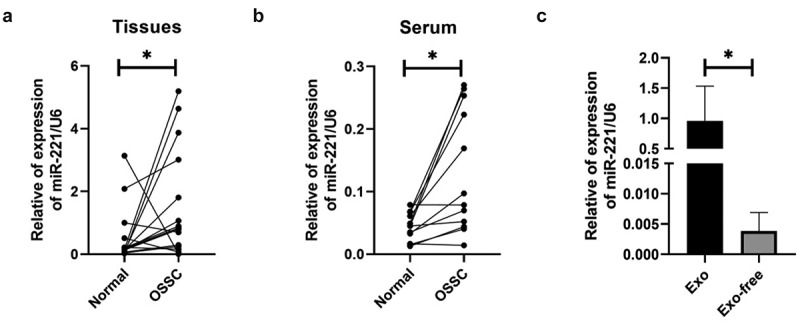
Elevated miR-221 expression in OSCC

MiR-221 expression levels in OSCC tissues, serum of patients were monitored with qRT-PCR. Figure 2a shows that miR-221 expression increased in OSCC tissues in comparison to NC, and the difference was statistically significant. Figure 2b shows that miR-221 expression was drastically higher in OSCC patients’ peripheral blood serum compared with NC. After isolating and removing exosomes from OSCC cell lines using ultracentrifuge, miR-221 expression drastically declined in the Exo-free group in comparison to the Exo group. The above results suggested that miR-221 expression increased in OSCC, and may be present in the exosomes of OSCC cells.

MiR-221 expression levels in tissues of each group (a), and peripheral blood serum (b) were examined by qRT-PCR; (c) MiR-221 expression level in groups, after ultra-centrifuging to remove exosomes, was detected by qRT-pCR. *P < 0.05.

### Exosome-mediated MiR-221 negatively regulated PIK3R1 expression

**Figure 3.**
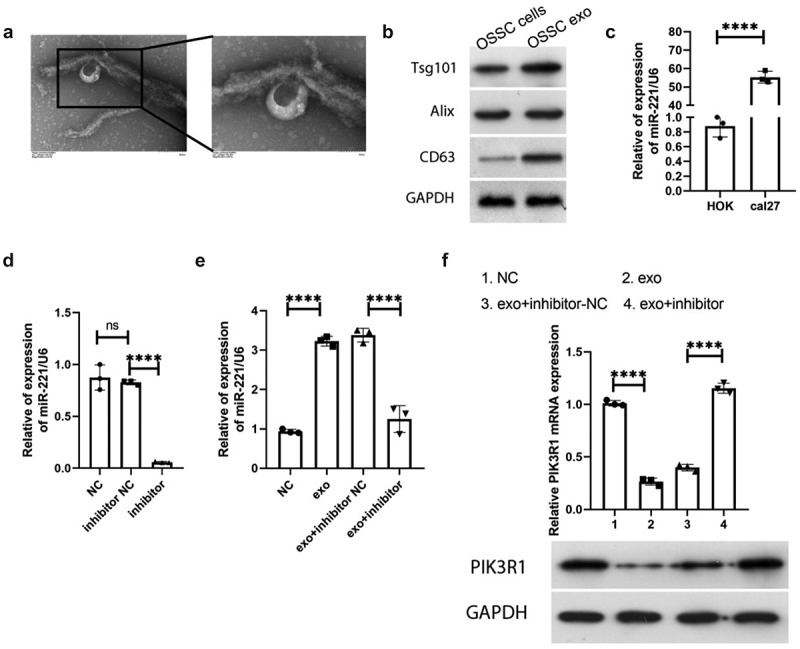
Exosome-mediated miR-221 negatively regulated PIK3R1 expression

After exosomes were obtained by ultracentrifugation, they were identified by electron microscopy and exosome membrane-specific protein electrophoresis, respectively. Electron microscopy showed that the exosomes were double-layered membrane structures (Figure 3a), with a size of about 100 nm. The specific membrane proteins tsg101, Alix, and CD36 all presented positivity (Figure 3b), indicating that the extracted isolated vesicular material was an exosome. The expression of miR-221 in human normal oral epithelial keratinocyte cell line HOK as well as human tongue squamous cell carcinoma cell line cal27 was detected by qRT-PCR, and the results of Figure 3c demonstrated that miR-221 was drastically upregulated in cal27 cells compared with HOK. The transfection efficiency of miR-221 inhibitor was confirmed by qRT-PCR, and Figure 3d manifested that no significant difference was manifested in miR-221 expression in inhibitor NC compared with NC (P > 0.05); miR-221 expression was significantly downregulated in the inhibitor group in comparison to inhibitor NC (*P* < 0.0001), indicating that miR-221 inhibitor transfection efficiency was good and could be used for subsequent experiments. After co-culture of HOK and cal27 with different transfection groups, miR-221 expression level in HOK of each treatment group were detected by qRT-PCR, and as shown in Figure 3e, miR-221 was significantly more expressive in the exo group in comparison to NC; miR-221 was noticeably less expressive in the exo + inhibitor group in comparison to exo + inhibitor-NC. The expression changes of PIK3R1 in HOK of each treatment group were monitored by qRT-PCR and western blot. figure 3f showed that PIK3R1 mRNA and protein expression in the exo group declined compared with NC; the mRNA and protein expression of PIK3R1 in the exo + inhibitor group drastically elevated comparing to exo + inhibitor-NC. The above results illustrate that exosomal miR-221 in OSCC cells negatively regulates PIK3R1 expression.

(a) Bilayer membrane structure of exosome (size~100 nm) under TEM; (b) western blot was used to detect the exosome-specific membrane proteins Tsg101, Alix, and CD63; (c) qRT-PCR was applied to monitor miR-221 expression in HOK and cal27; (d and e) qRT-PCR was applied to monitor miR-221 expression level in HOK cells after co-culture with cal27 from different treatment groups; (f) qRT-PCR (upper) and western blot (bottom) were used to examine the expression change of PIK3R1 in HOK cells following co-culture with cal27 from different treatment groups. ****P < 0.0001.

### MiR-221 targeted and negatively regulated PIK3R1

**Figure 4.**
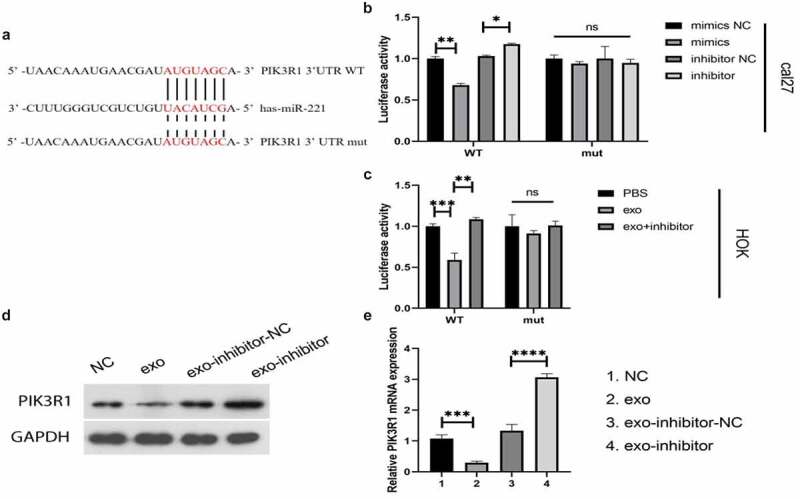
MiR-221 targeted and negatively regulated PIK3R1

MiR-221 was found to negatively regulate PIK3R1 expression. We speculated that there may be targeted regulation between the two; the dual-luciferase reporter system verified the conjecture. Figure 4a shows a prospective binding site between miR-221 and PIK3R. In the cal27 cell line (Figure 4b), co-transfection of PIK3R1 3′UTR WT with mimics or inhibitors enhanced or decreased the fluorescence intensity of PIK3R1 3′UTR, respectively; however, the fluorescence intensity of PIK3R1 3′UTR did not change in groups of PIK3R1 3′ UTR mut co-transfected with mimics and inhibitor compared with their respective controls. In HOK cells (Figure 4c), exo from cal27 significantly decreased the fluorescence intensity of PIK3R1 3′UTR compared with PBS; exo + inhibitor significantly increased the fluorescence intensity of PIK3R1 3′UTR compared with exo. The results of western blot (Figure 4d) and qRT-PCR (Figure 4e) further verified the targeted and negative regulatory relationship between miR-221 and PIK3R1: both mRNA and protein expression of PIK3R1 significantly decreased in cells from the exo groups relative to NCs; PIK3R1 mRNA and protein expression noticeably elevated in cells from the exo-inhibitor groups compared with exo-inhibitor-NCs. The above results concluded that miR-221 can target and negatively regulate PIK3R1.

(a) Prospective binding site between miR-221 and PIK3R1; (b and c) Dual-luciferase assay tested for the target binding ability between miR-221 and PIK3R1; (d) (western blot) and (e) (qRT-PCR) were applied to monitor the changes in PIK3R1 protein and mRNA expressions in different treatment groups. *P < 0.05, **P < 0.01, ***P < 0.001, ****P < 0.0001.

### OSCC-derived exosomal miR-221 promoted HUVEC migration

**Figure 5.**
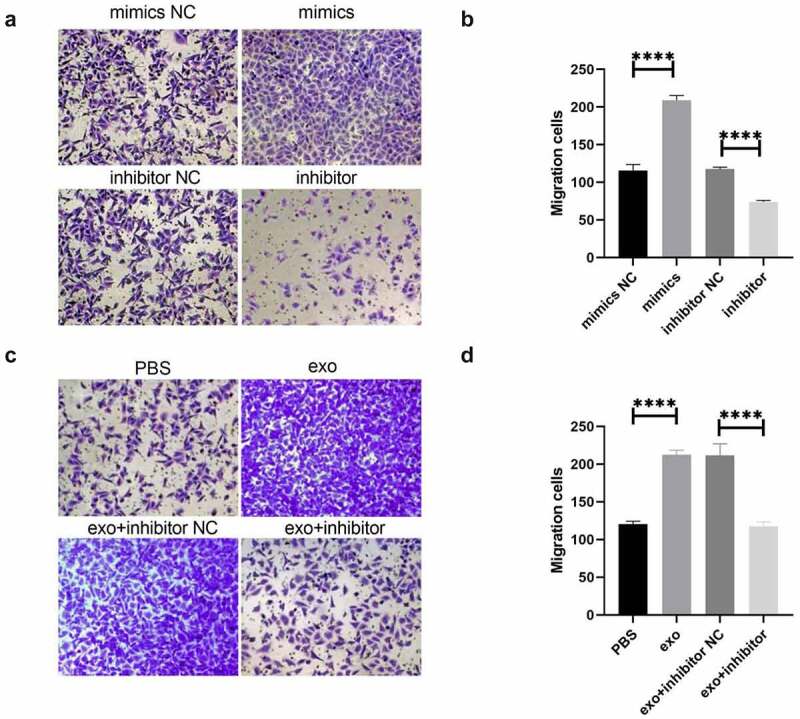
OSCC-derived exosomal miR-221 promotes HUVEC migration

Vascular endothelial cell migration and tube formation were important processes in tumor-related abnormal blood vessel formation. Figure 5a-b shows that HUVEC tube migration was significantly enhanced in the mimics group in comparison to mimics NC; migration was drastically attenuated in the inhibitor group comparing to inhibitor NC. Figure 5c-d shows that HUVEC migration was drastically enhanced in the exo group compared with PBS; HUVEC tube formation migration was significantly attenuated in the exo + inhibitor group comparing to exo + inhibitor NC. These outcomes suggested that OSCC-derived exosomal miR-221 can promote HUVEC migration.

(a and b) After the overexpression/inhibition of miR-221 was investigated by Transwell, the change in HUVEC migration ability; (c and d) The effect of exosomal miR-221 on HUVEC migration monitored by Transwell. ****P < 0.0001.

### OSCC-derived exosomal miR-221 promotes HUVEC tube formation

**Figure 6.**
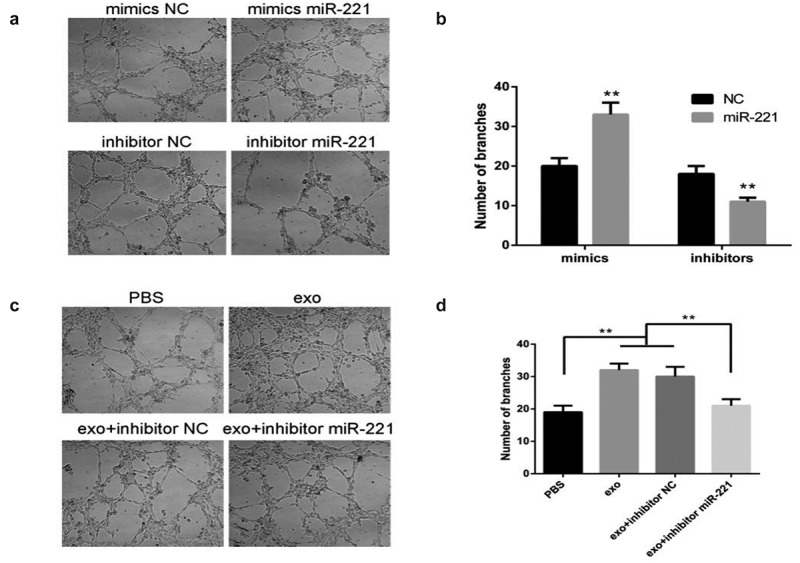
OSCC-derived exosomal miR-221 promotes HUVEC tube formation

Figure 6a-b illustrates that the HUVEC tube formation ability of mimics group was drastically enhanced comparing to mimics NC; the HUVEC tube formation ability of inhibitor group was significantly attenuated compared with inhibitor NC. The results of Figure 6c-d show that HUVEC tube formation ability was noticeably enhanced in the exo group relative to PBS; HUVEC tube formation ability was noticeably attenuated in the exo + inhibitor group relative to exo + inhibitor NC. These results illustrate that OSCC-derived exosomal miR-221 could promote HUVEC migration and tube formation, leading to angiogenesis.

(a and b) After the overexpression/inhibition of miR-221 was investigated by tube formation assay, the change in HUVEC tube formation ability; (c and d) The effect of exosomal miR-221 on HUVEC tube formation observed by assay. **P < 0.01.

## Discussion

Among head and neck malignancies, OSCC has one of the highest incidence rate. Studies have found that some patients with advanced OSCC even with complete surgical resection had a survival period of less than 30 months [17]. In addition, the patients’ 5-year survival rate is also related to the location and progression of the tumor, age and underlying disease of the patient. Therefore, for OSCC, finding tumor markers for molecular diagnosis, prediction of prognosis, and targeted therapy is important for the treatment of tumors. There is high clinical value in understanding the mechanism of occurrence, progression, invasion and metastasis of OSCC and in revealing the oncogenes and tumor suppressor genes of OSCC, all of which are conducive to improve the treatment of OSCC. In this study, we observed that PIK3R1 was downregulated in OSCC tissues, suggesting that PIK3R1 may be a prognostic factor for OSCC and could be a potential therapeutic target.

Recent studies have shown that the tumor microenvironment (TME) has an important promoting effect on the development of OSCC [18,19]. Extracellular matrix remodeling, immunosuppression, angiogenesis, and altered metabolism of energy substances that occur within the TME provide more favorable conditions for the development and progression of tumors [20,21]. The TME is a special local environment formed by the long-term interaction between tumors and surrounding stroma, as well as various cells, including fibroblasts, macrophages, adipocytes and other benign cells and extracellular matrix (ECM), signaling molecules and new vascular structures [22]. Angiogenesis is a fundamental characteristic of tumor, which refers to abnormal angiogenesis to support tumor growth and metastasis.

More and more studies report that tumor cell-released exosome is a decisive factor in vascular cell activation during oncogenesis, e.g., exosomes derived from melanoma (B16-F10), TNBC (Hs578T) and hepatocellular carcinoma (HepG2) cell lines [23]. Exosomes offer a relatively stable space for the selected therapeutic agents: they could probably improve cell-specific homing, and fuse with plasma membrane so that the drug can enter the cell directly. Allogeneic exosomes are thought to suppress the immune response of host cells and have the potential to overcome the drawbacks of the immune response brought about by cell therapy [9,24]. Therefore, there are great prospects for studying and engineering exosomes. In this study, we found that exosomes secreted by OSCC cells contained miR-221; upregulated miR-221 can target and negatively regulate PIK3R1 expression, promote HUVEC migration and tube formation, thus leading to angiogenesis.

Although studies are investigating the direct modification of exosomes [25–27], the most common approach currently is to engineer the mother cell, which secretes modified exosomes with therapeutic values. The key of this therapeutic approach is to understand the factors that impact the release and uptake of the modified exosome content. In 1998, when it was found that dendritic cell (DC)-derived exosomes release T cells and stimulate tissue major histocompatibility complex (MHC) I and II, DC-derived exosomes were used in cell-free vaccine for the first time [28]. Hepatoma carcinoma cell-derived exosomal miR210 might enter endothelial cells, and affect SMAD4 and STAT6 to promote tumor angiogenesis [29].

## Conclusion

Taken together, our data suggest that OSCC-derived exosomal miR-221 promotes HUVEC migration and angiogenesis by targeting PIK3R1 in OSCC. The findings suggested that miR-221 and PIK3R1 can potentially become therapeutic targets for clinical treatment of OSCC.

## Highlights

1. PIK3R1 expression was decreased and miR-221 expression was elevated in OSCC.
2. MiR-221 targeted and negatively regulated PIK3R1.
3. OSCC-derived exosomal miR-221 promoted HUVEC migration and tube formation.
